# Effects of Manual Therapy on Respiratory Function in Healthy Individuals: A Randomized, Sham-Controlled Clinical Trial of Efficacy and Safety

**DOI:** 10.3390/healthcare14142051

**Published:** 2026-07-08

**Authors:** María Isabel Rocha-Ortiz, Jesús Sánchez-Más, Sonia del Río-Medina, Sergio Montero-Navarro, María Teresa Pérez-Gracia, Jaume Morera-Balaguer

**Affiliations:** 1 Department of Physiotherapy, School of Health Sciences, Universidad Cardenal Herrera-CEU, CEU Universities, 03204 Elche, Alicante, Spainsergio.montero@uchceu.es (S.M.-N.); jmorera.el@uchceu.es (J.M.-B.); 2 Department of Biomedical Sciences, School of Health Sciences, Universidad Cardenal Herrera-CEU, CEU Universities, 03204 Elche, Alicante, Spain; jesus.sanchez@uchceu.es; 3 Department of Pharmacy, Institute for Biomedical Sciences, School of Health Sciences, Universidad Cardenal Herrera-CEU, CEU Universities, 46115 Alfara del Patriarca, Valencia, Spain

**Keywords:** manual therapy, spirometry, respiratory disease

## Abstract

**Background**: Chronic respiratory disease is currently a global health problem. Secondary problems, such as skeletal muscle disorders, among which cervical spine and diaphragm dysfunctions are two of the most common, can affect the lung function of these subjects. Manual therapy could be a suitable treatment for these problems. However, although this is a very broad area of research, there is no consensus on its usefulness. **Objectives:** To investigate the effects of the diaphragmatic stretch technique and the high-speed, low-amplitude C3–C4 rotational thrust technique, applied alone or in combination, on respiratory function in healthy adults. Peak expiratory flow (PEF) was the primary outcome; forced vital capacity (FVC) and forced expiratory volume in 1 s (FEV1) were secondary outcomes included to assess safety. **Methods**: A randomized, sham-controlled clinical trial with blinded outcome assessment with 152 healthy participants distributed in four groups was conducted: Group 1 subjects underwent the Diaphragmatic Stretch Technique, Group 2 subjects underwent the high-speed, low-amplitude thrust technique in C3–C4 rotation, Group 3 underwent both techniques, and Group C (control group) received a simulation of one of the two techniques. The three outcome variables were recorded immediately after the intervention and 5 min after. Study registered on ClinicalTrials.gov (NCT03732222). **Results**: The results show a significant increase in PEF for Group 1, visible in the immediate post-intervention and at 5 min post-intervention. Group 3 also showed a significant increase in PEF, although only at 5 min post-intervention. **Conclusions**: High-speed, low-amplitude C3–C4 rotation thrust technique and Diaphragmatic Stretch Technique can improve the PEF in healthy subjects.

## 1. Introduction

Chronic respiratory disease is currently a global health problem. For example, Chronic Obstructive Pulmonary Disease (COPD) is the most common noncommunicable respiratory disease, affecting approximately 300 million people, or 4% of the world’s population [1,2]. Worldwide, it is the third most common cause of death [3,4].

Pharmacological intervention is an essential component of the treatment of these pathological conditions; however, it must be complemented by an appropriate pulmonary rehabilitation protocol (PR) [5,6]. Thus, PR is a multi-disciplinary approach that includes patient assessment, exercise training, health education, nutritional intervention, and psychosocial support [6,7,8]. Although there is evidence that PR can enhance exercise capacity, there is no consensus on whether it can deliver any clinically meaningful increases in lung function [9,10,11,12].

Some authors consider that a significant proportion of COPD morbidity arises from secondary problems such as cardiac deconditioning, skeletal muscle disorders, and anxiety [4,13]. Among the musculoskeletal disorders, cervical spine dysfunction and diaphragm impairment are particularly relevant because they directly affect respiratory mechanics. There is also growing evidence of the reverse relationship: chronic neck and back pain, often associated with postural dysfunction, can negatively influence diaphragmatic function and, consequently, pulmonary performance [14,15,16]. Patients with chronic neck pain exhibit reduced lung volumes, decreased chest mobility, and lower respiratory muscle strength [17,18,19,20,21,22]. Diaphragm dysfunction, characterised by atrophy and reduced endurance, impairs effective ribcage expansion, increases the work of breathing, and diminishes functional capacity [23,24,25,26,27]. These alterations are closely linked to postural changes and altered thoracic mechanics, creating a bidirectional interaction between musculoskeletal and respiratory systems. These observations provide the pathophysiological rationale for investigating manual therapy techniques specifically targeting the cervical spine and diaphragm. Given this bidirectional relationship between cervical/thoracic musculoskeletal function, diaphragmatic performance and respiratory outcomes, manual therapy techniques directed at these structures have been proposed as a potential therapeutic approach.

The effects of manual therapy on respiratory function constitute a very broad area of research. However, there is no consensus among authors regarding its efficacy. Studies examining osteopathic manual treatment (OMT) on respiratory function display considerable heterogeneity in terms of treatment protocols, treatment duration, outcome data collection and population characteristics. Techniques are frequently applied in combination, either as a single session or over multiple sessions, and often alongside pulmonary rehabilitation [8,28], physical exercise [8,29,30], or respiratory muscle training [31]. Positive results are commonly attributed to synergistic effects. Research has predominantly focused on individuals with COPD [28,32,33,34,35], although trials have also been conducted in children and adults with asthma [36] and cystic fibrosis [37]. Lung function has been evaluated using spirometry [33,34,35,36,37,38], respiratory muscle strength testing [31], quality-of-life questionnaires and other subjective outcomes [8,35,37], blood gas analysis [39,40], and functional assessments such as the 6 min walk test [8,29,31,33,34,35,39].

A systematic review published in 2019 [32] concluded that OMT applied in isolation yields transient improvements in respiratory rate, heart rate, and symptoms, whereas findings when combined with exercise therapy are mixed and appear technique-dependent. A more recent review (2024) [41] identified only five studies of adequate quality examining manual therapy effects on respiratory system function; all but one demonstrated statistically significant differences favouring OMT over control conditions.

However, relatively few studies have examined the influence of these techniques on respiratory parameters in healthy subjects [38,41,42]. Prior to applying any technique to individuals with pathology, it is necessary to characterise its physiological effects on relevant parameters in healthy volunteers. This preliminary step ensures safety, feasibility, and reproducibility, in accordance with international ethical standards [43,44]. Conducting the study in healthy participants also permits control of confounding variables inherent to pulmonary disease, optimisation of the experimental design, and establishment of reference physiological values. The findings can then inform the design of subsequent trials in clinical populations. We therefore consider this phase essential before progressing to patients with respiratory pathology.

Demonstrating short-term changes in healthy individuals is clinically meaningful for several reasons. First, it furnishes direct evidence of the immediate biomechanical and neuromuscular effects of the selected manual therapy techniques on respiratory function, unconfounded by disease-specific factors. The statistically significant increases in peak expiratory flow (PEF) observed immediately and at five minutes post-intervention indicate that these techniques can rapidly modulate airflow, an effect that may prove beneficial in clinical scenarios where patients have restricted capacity for active participation or during acute respiratory events. Second, the lack of adverse effects on fundamental spirometric indices (FVC and FEV1) in healthy subjects supports the safety of these interventions when delivered in a single session. Third, this sequential, low-risk investigative strategy, beginning with asymptomatic volunteers. conforms to established ethical principles for the development of therapeutic interventions. Finally, even transient physiological responses in healthy individuals can guide the integration of manual therapy into rehabilitation protocols as a low-burden adjunct capable of enhancing immediate respiratory function or improving tolerance to subsequent exercise-based components of pulmonary rehabilitation. We did not anticipate large improvements in static lung volumes (FVC and FEV1) in healthy young adults with normal respiratory function, as these parameters are already close to their physiological maximum. These variables were nevertheless included to comprehensively evaluate the safety of the techniques and to determine whether any detrimental effects on overall lung function could occur. However, some studies, such as those by Stepnik et al. [38] and Küçük et al. [45], reported a significant increase in PEF following osteopathic manual techniques in young, healthy adults. Furthermore, some studies demonstrate multiple benefits resulting from improved PEF1, including improved airflow obstruction in subjects with asthma or COPD. These findings reinforce our rationale for designating PEF as the primary outcome, as it appears to be a more responsive marker of immediate biomechanical and neuromuscular effects in our sample following these techniques than traditional static spirometric volumes.

In contrast, we hypothesised that more dynamic parameters, such as PEF, might be more sensitive to immediate changes in thoracic mobility and respiratory muscle function even in asymptomatic individuals.

Manipulation techniques directed at the cervical and/or thoracic spine together with diaphragmatic release are frequently employed in studies investigating the effect of OMT on breathing [33,38,45,46,47,48,49,50,51,52]. Nevertheless, in the majority of published protocols these techniques form part of a larger therapeutic package [33,47,48,49,53]. Based on the above rationale, we were particularly interested in determining whether the isolated application of these two specific techniques, high-velocity, low-amplitude rotational thrust at C3–C4 and diaphragmatic stretch, produces measurable effects on respiratory function when applied independently of other interventions. We deliberately chose to evaluate these two specific techniques in healthy individuals without pre-existing cervical or diaphragmatic dysfunction in order to determine their direct physiological effects on respiratory function, independent of any underlying impairment. Studying asymptomatic volunteers allows us to isolate the biomechanical and neuromuscular actions of the techniques while minimising confounding variables commonly present in clinical populations, such as altered muscle tone, pain inhibition, postural compensations, or pre-existing changes in respiratory mechanics. This approach also aligns with international ethical standards for the development of manual therapy interventions, which recommend first establishing safety and physiological effects in healthy individuals before progressing to patients who may present contraindications or different responses. Although the magnitude of change was expected to be modest in this population, the study provides valuable reference data on the direction, variability, and effect size of acute responses in PEF, FVC, and FEV1. These data can inform the design and sample size estimation of future trials in clinical populations, where larger or more clinically meaningful effects may be observed.

We hypothesised that minimising both the number of techniques and total treatment duration could enhance clinical applicability and resource efficiency in healthcare settings subject to time and staffing constraints. Accordingly, the present study was designed to elucidate the isolated effects of these two manual therapy techniques on FVC, FEV1, and PEF in healthy individuals, thereby providing a theoretical and methodological foundation for future research in populations with respiratory disease. The immediate and 5 min post-intervention measurements were chosen to capture potential rapid physiological changes induced by the techniques, as this study was conceived as a preliminary step to investigate acute effects and safety in healthy individuals prior to future research involving multiple sessions or clinical populations.

## 2. Materials and Methods

### 2.1. Design

This was a randomized, sham-controlled clinical trial with blinded outcome assessment. Participants were not informed whether they received a real or simulated technique. The evaluator who performed the spirometric measurements was blinded to group allocation through coded assignment. The therapist administering the intervention was necessarily aware of the technique applied. Assignments were made by an external researcher using Epidat v4.2 and kept in a secure file. The control group received a simulated technique to avoid expectation bias.

### 2.2. Setting and Participants

Adult participants were recruited from CEU Cardenal Herrera University via emails and classroom announcements. Eligible participants were healthy men and women without current respiratory disorders (including obstructive and restrictor diseases), history of rib fractures, thoracic deformities, digestive problems, hepatitis, hepatobiliary lesions, heart disease and/or arterial hypertension, surgical scar in the abdomen, thorax and/or neck, cancer, use of local anaesthetics, pregnancy, diagnosed psychological imbalances, contraindication to any of the study techniques, active smoker. These exclusion criteria are related to contraindications for certain OMT techniques used in this study, as well as conditions that may alter lung function.

### 2.3. Recruitment

The participants were recruited from undergraduate students at the University Cardenal Herrera CEU de Elche (Alicante). The population of this centre represents the heterogeneity of profiles necessary to carry out this study. The stages of selection are shown in.

### 2.4. Study Procedure

The study was approved for human subjects research by the Research Ethics Committee of the Cardenal Herrera CEU University (CEI13/01). This study was also registered on ClinicalTrials.gov as a clinical trial (20 November 2018, NCT03732222, https://clinicaltrials.gov/study/NCT03732222 (accessed on 2 July 2026)). The study was registered after participant recruitment had begun, at a time when this practice was not yet common in physiotherapy in Spain. Ethical approval and informed consent were ensured from the outset, and the registration was later completed to strengthen transparency and ensure compliance with international research standards. Eligible participants were selected based on the inclusion and exclusion criteria. The purpose of the study was made clear to each participant, and written informed consent and personal data were obtained prior to participation in the study. The participants were randomly assigned to the four study groups using the Epidat v4.2 statistical program, obtaining four subsamples of the same size without replacement, with the same probability of inclusion. Each subject was assigned an identification code, following an ordered numbering from 1 to 152, to ensure anonymity.

Baseline assessments were performed after randomization and written informed consent. These included demographic characteristics (age and biological sex by self-report), anthropometric measurements, self-reported physical activity levels, and baseline spirometric parameters. Weight was measured to the nearest 0.1 kg using a calibrated digital scale with participants wearing light clothing and no shoes. Height was measured to the nearest 0.1 cm using a wall-mounted stadiometer. Body mass index (BMI) was calculated as weight in kilograms divided by height in meters squared. Chest circumference was measured at the level of the xiphoid process using a non-stretchable tape measure at the end of a normal expiration. Frequency and intensity of physical activity were assessed via a short structured self-report questionnaire using the categorical responses subsequently presented in Table 1 (0 h, 1–5 h or >5 h per week; none, light-moderate or intense).

Prior to each spirometric assessment (baseline, immediate post-intervention and 5 min post-intervention), participants rested quietly in a seated position for at least 10 min to avoid fatigue effects [54,55]. All spirometric assessments (FVC, FEV1 and PEF) were conducted by a single trained evaluator who remained blinded to group allocation through coded participant identification. A portable spirometer (Datospir 120, Sibelmed, Barcelona, Spain) was used after daily calibration according to the manufacturer’s instructions. Testing was performed with participants seated upright and wearing a nose clip, strictly following the 2019 ATS/ERS standardization of spirometry [56,57]. A minimum of three (and up to eight) maximal forced expiratory maneuvers were performed after maximal inspiration until three acceptable and reproducible maneuvers were obtained (difference between the two largest values for FVC and FEV1 ≤ 150 mL or ≤5%). The highest values from acceptable maneuvers were recorded for analysis.

To evaluate safety, participants were systematically questioned immediately after the intervention and at the 5 min follow-up regarding any adverse events or sensations (pain, dizziness, dyspnea, chest discomfort or other symptoms). In addition, any post-intervention spirometric decline >15% from baseline was predefined as a potential safety concern for clinical review.

Thereafter, Group 1 subjects underwent the Diaphragmatic Stretch Technique, performed with the subject in a supine position and knees flexed. The therapist placed their hands on the costal margins and, during inspiration, applied a cephalad traction while maintaining the stretch during expiration. Ten repetitions were performed. Group 2 participants received a high-velocity, low-amplitude thrust technique at the C3–C4 level in rotation, with the subject positioned supine. The therapist stabilized the target vertebra with one hand while placing the other at the cranial point, adjusting the levers (chin tuck, neutral flexion–extension, ipsilateral lateral flexion, and contralateral rotation) prior to delivering a contralateral rotational thrust. Group 3 underwent both techniques. Group C (control group) underwent a simulated intervention consisting of the subject lying supine with a cushion under the head while the therapist, positioned at the head of the table, placed their hands on the costal margins without applying any movement, for one minute, to rule out stimulation effects. The three outcome variables were recorded immediately after the intervention and 5 min after.

All interventions were performed by the same licensed physiotherapist (M.I.R.O.), a member of the research team with more than fifteen years of clinical experience in manual therapy and osteopathic manipulative techniques. Prior to the beginning of the study, the therapist completed specific training to standardise the application of both the diaphragmatic stretch technique and the high-velocity, low-amplitude C3–C4 rotational thrust technique. Interventions were delivered strictly according to the standardised protocols described in the Foundations of Osteopathic Medicine [58]. The use of a single experienced therapist ensured consistency in technique delivery across all participants.

### 2.5. Description of Outcome Measures

The dependent variables included forced vital capacity (FVC), forced expiratory volume in one second (FEV1), and peak expiratory flow (PEF). The primary outcome measure was PEF. This dynamic parameter was pre-specified as primary because it is particularly sensitive to immediate changes in thoracic mobility, respiratory muscle coordination, and neuromuscular drive induced by manual therapy techniques, even in healthy individuals with near-maximal baseline lung function.

The secondary outcome measures were FVC and FEV1, together with safety monitoring (occurrence of adverse events or symptoms and any post-intervention decline >15% from baseline in any spirometric parameter). FVC and FEV1 were included as secondary endpoints to provide a comprehensive safety evaluation by confirming the absence of any detrimental effect on static lung volumes.

The independent variables were gender (male/female), age, weight, height, body mass index, chest circumference, and the frequency and intensity of sports activity.

### 2.6. Sample Size Estimation

A pilot study was conducted which involved 40 individuals who met the inclusion criteria and who were randomly assigned to the four intervention groups of equal size. According to the data obtained from the pilot study, the adequate sample size for a confidence level α of 0.05 and a power of 80% in a one-factor repeated measures ANOVA design with four levels was 152 individuals (38 per group). The sample size was calculated using the G*Power v3.1 program.

### 2.7. Data Analysis

The Kolmogorov–Smirnov test was used to determine whether the data of the variables followed a normal distribution. Quantitative variables were shown as mean ± standard deviation and 95% confidence intervals. Categorical variables were shown as number (percentage) and frequency tables. For the analysis of population characteristics and baseline spirometry data, the U-Mann–Whitney test was used to compare quantitative variables and the Chi2 test was used to compare qualitative variables. For the analysis of the spirometry data at follow-up, the Wilcoxon test was used to compare the equality of means between the results obtained at follow-up and the baseline mean, and the Friedman test was used to analyse intragroup changes between the three measurement times. The differences between the groups were analysed by comparing the change observed from baseline to immediately after the intervention (Δ1 = Post − Pre) or 5 min after the intervention (Δ2 = 5 min Post − Pre). The effect size was calculated using Cohen’s d. Statistical significance was assumed at *p* < 0.05. Statistical analysis was performed using the IBM SPSS Statistics software program for Windows, version 29.0 (SPSS Inc., Chicago, IL, USA).

## 3. Results

**Figure 1:**
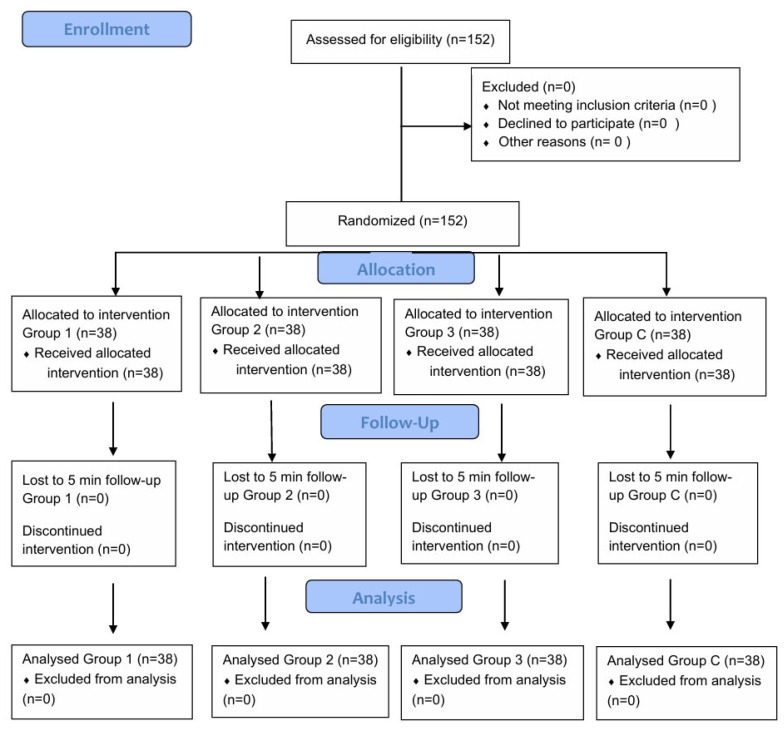
CONSORT 2025 flow diagram.

We selected 152 healthy participants. There were no dropouts during the study. Finally, a total of 152 patients, as per the sample size, were included (Figure 1). No adverse events or discomfort were reported by any of the 152 participants during the intervention or at the follow-up assessments. Furthermore, no clinically significant declines (>15% from baseline) in FVC, FEV1 or PEF were observed in any group.

Baseline characteristics of the patients are presented in Table 1. Most of the participants were women (59.2%), with a mean age of 22.6 years and normal weight. Most reported physical activity between 1 and 5 h with moderate intensity. Forced spirometry values were within the normal range. There were no significant differences in the baseline values of any of the anthropometric characteristics or in any of the respiratory variables between the different groups.

Differences between values for the 3 study variables (FVC, FEV1 and PEF) pre-intervention, post-intervention and at 5 min post-intervention are summarized in Table 2. The results show a significant increase in PEF for the group combining stretching and rotation, visible in the immediate post-intervention and at 5 min post-intervention. The group subjected only to rotation also showed a significant increase in PEF, although only at 5 min post-intervention.

The remaining respiratory variables did not show significant differences for any of the treatments in the intragroup analysis. Similar results were obtained in the intergroup analysis (Table 3). The respiratory variables FVC and FEV1 showed no significant differences between the intervention groups and the control group when comparing the change observed from baseline to immediately post-intervention (Δ1) or 5 min after intervention (Δ2). However, the results show a significantly greater increase from baseline immediately after the intervention in the group that combined stretching and rotations compared to the control group.

Table 3 evaluates the spirometry data according to gender for each of the intervention groups. In women, both the rotation group and the rotation + stretching group showed a significant increase in PEF in the immediate post-intervention period, which was maintained at 5 min post-intervention. This significant increase in PEF was not observed in the male group. The remaining variables (FVC and FEV1) did not show significant differences for any of the treatments when analysed by gender.

## 4. Discussion

The main purpose of our study was to investigate whether the diaphragmatic stretch technique and the high-speed, low-amplitude C3–C4 rotational thrust technique, applied together, affect three pulmonary indicators: FVC, FEV1 and PEF.

We found that there was a statistically significant improvement in PEF in the group receiving only the high-speed, low-amplitude C3–C4 rotational thrust technique (G2) and in the group receiving both interventions (G3) versus the group that received only the Diaphragmatic Stretch Technique (G1) and the control group (GC). In the case of G2, we observed that this improvement was only significant at the 5 min measurement. However, in the post-intervention measurement we found a trend, although not significant. Nonetheless, the fact that it occurred in asymptomatic healthy subjects means that in individuals with pathologies a significant variation may perhaps be found.

These findings are consistent with those reported by other researchers in the field. Martínez González MA reported that the high-speed, low-amplitude rotational thrust technique at C3–C4 has a positive influence on PEF in healthy patients, both post-intervention and at 5 min, with no influence on other spirometry variables [53]. Stepnik et al. [38] conducted a study with 30 women between 19 and 46 years old, to whom they applied OMT thoracic thrust (manipulations of vertebral joints and ribs), the sternal pump technique and stretching of the diaphragm, finding that these osteopathic techniques exert an influence on PEF in healthy individuals; however, it does not affect FVC and FEV1. The conclusion was that osteopathic techniques do not seem to improve lung health, as reflected in FEV1 and FVC; however, they improve the respiratory function aspects reflected by PEF in the participants without any history of lung disease.

The observation that a single application of these two techniques produced statistically significant improvements in PEF is both mechanistically and clinically relevant. Manual therapy techniques targeting the cervical spine and diaphragm can rapidly influence respiratory mechanics through several immediate pathways: increased thoracic mobility and rib cage compliance, enhanced diaphragmatic excursion, reduced myofascial tension in accessory respiratory muscles, and possible modulation of autonomic nervous system activity. These acute biomechanical and neuromuscular changes are particularly well captured by dynamic parameters such as PEF, which is known to be sensitive to small alterations in expiratory muscle function and airway resistance even in the absence of changes in static lung volumes.

The differential response across groups is also noteworthy. The lack of a statistically significant change in the diaphragmatic stretch group alone (G1) suggests that, in young healthy individuals with already optimal baseline diaphragmatic function and thoracic compliance, this technique in isolation may not produce a sufficiently large additional effect on expiratory flow to reach statistical significance within the acute timeframe examined. In contrast, the high-velocity, low-amplitude C3–C4 thrust (G2) appears to introduce a distinct neuromodulatory component, likely via cervical proprioceptive input and possible influence on phrenic nerve outflow, that more readily translates into measurable improvement in PEF, albeit with a slightly delayed time course (significant only at 5 min). The combination of both techniques (G3) produced the earliest and most sustained effect, consistent with a synergistic action on thoracic mechanics and neuromuscular control. These patterns support the notion that different manual therapy techniques may engage partially distinct physiological pathways with different temporal profiles, even when targeting the same functional outcome.

Our findings are consistent with previous single-session studies demonstrating immediate or short-term improvements in PEF or other respiratory parameters following osteopathic or manual therapy interventions in healthy individuals [38,50,53]. This pattern is consistent with previous high-quality studies in healthy populations, which have similarly shown selective improvements in PEF without concomitant changes in FVC or FEV1 following manual therapy interventions [38,45]. From a clinical standpoint, the ability of a brief, single-session intervention to produce a detectable change in expiratory flow supports its potential value as a low-burden adjunct within pulmonary rehabilitation programmes. This may be especially useful in populations with limited exercise tolerance, acute respiratory exacerbations, or during the initial phases of rehabilitation when patients may not yet tolerate multiple or prolonged sessions. Although the duration of these acute effects was not assessed beyond five minutes, the demonstration of a measurable physiological response after only one application provides proof-of-concept and a mechanistic rationale for future studies investigating cumulative effects with repeated sessions or therapeutic benefits in clinical populations with respiratory impairment.

This result is interesting, since different authors have highlighted the importance of PEF as an independent predictor of health status, and physical and cognitive functions [58], hospitalization, frailty development [59], mortality from respiratory and other causes in the elderly [60,61,62,63]. PEF is a highly reliable test to estimate airflow obstruction, which is very important for the screening and treatment of asthma and COPD, especially for adults and the elderly [64,65,66,67].

Although the absolute increases in PEF were modest, they reached statistical significance in two of the intervention groups and are consistent in magnitude with those reported in previous single-session manual therapy studies conducted in healthy individuals [38,50,53]. In asymptomatic young adults with normal baseline lung function, even relatively small acute improvements in a dynamic parameter such as PEF can be considered physiologically meaningful, as they reflect immediate enhancements in expiratory muscle coordination, thoracic mobility and neuromuscular drive. Importantly, these changes occurred without any accompanying deterioration in FVC or FEV1, reinforcing the safety profile of the interventions. While the minimal clinically important difference for PEF has not been firmly established in healthy populations, the observed effects provide proof-of-concept that these techniques can rapidly modulate respiratory mechanics in asymptomatic individuals. Whether comparable effects occur in clinical populations with respiratory or musculoskeletal conditions, and whether they translate into meaningful benefits for patients, requires specific investigation in future studies.

As anticipated, we did not observe significant changes in FVC or FEV1. This finding was expected in healthy individuals with normal lung function and confirms that the techniques do not produce detrimental effects on static lung volumes. In contrast, the significant improvements observed in PEF support our hypothesis that dynamic expiratory flow parameters may be more responsive to the immediate biomechanical effects of these manual therapy techniques. This allows us to ensure that the application of these two techniques is not detrimental in terms of the variables studied and can therefore be applied in individuals with pathology. We must bear in mind that since FVC and FEV1 are two variables that indicate the general state of the respiratory system, it is logical that in healthy patients there is no evident improvement. Lorenzo s et al. [68] applied OMT techniques to 53 healthy university students, analysing, among others, the influence of each of them on FEV1, FVC, and FEV1/FVC. They did not obtain statistically significant improvements in any of the parameters.

González-Álvarez et al. [50], in a study with 80 healthy volunteers from the Faculty of Health Sciences, found that diaphragm stretching improves pulmonary function in healthy adults in a short period, with a significant increase in FVC and FEV1 after the technique and at 5 min, although with a decrease at 20 min in FEV1. However, the study sample differed from ours. The mean age was higher (36.33 ± 15.93) in their intervention group and 37.40 ± 15.82 in the placebo group, compared to 22.5 ± 5.7 in the overall sample in our case. Furthermore, smokers were accepted in their study (51.16% of the intervention group sample, and 51.35% of the placebo group), provided they did not report a consumption of 20 cigarettes a day. This seems to suggest that such techniques may improve lung health outcomes when there are factors that indicate a non-significant loss of lung capacity. Several authors have shown that manual therapy can mitigate the effects of age-related changes in lung capacity loss even when it is not pathological.

Our results showed that changes in PEF occur only in women, with no significant differences in men. This result could be explained by anatomical and biomechanical differences in the cervical spine of women, such as greater flexibility, lower muscle mass and higher baseline cervical proprioceptive acuity, which may render them more responsive to the neuromodulatory effects of high-velocity thrust techniques [69,70]. In addition, sex-related variations in connective tissue compliance and hormonal influences on muscle tone and joint laxity could facilitate greater immediate changes in thoracic mechanics following manual intervention. These differential responses warrant further investigation in larger, sex-balanced samples to determine whether they reflect true physiological differences or are influenced by other factors such as baseline physical activity levels.

This study presents some limitations. Although the present study was a sham-controlled clinical trial with blinded outcome assessment, some participants were physiotherapy students and may have been aware of the techniques, which may have influenced their subjective assessment of respiratory change based on their possible pre-existing beliefs about OMT. A major limitation of this study is that the sample was composed exclusively of young, healthy university students. This characteristic significantly limits the external validity and generalizability of our findings to other populations, such as older adults or individuals with respiratory or musculoskeletal conditions. Although this population was intentionally selected to first evaluate the safety and physiological effects of the techniques in the absence of disease-related confounders, the results should be interpreted with caution when extrapolating to clinical populations. Future studies should include more heterogeneous samples, including older adults and patients with respiratory pathology, to confirm the applicability and clinical relevance of these interventions. Another limitation is that we did not formally assess the credibility of the sham intervention. Although participants were kept unaware of whether they received a real or simulated technique, future studies would benefit from including a credibility or expectancy questionnaire to further strengthen blinding integrity.

Additionally, outcome assessments were limited to the immediate post-intervention period and 5 min later. Although this short timeframe was appropriate to evaluate acute physiological effects and safety, it precludes any conclusions regarding the duration or clinical sustainability of the observed changes in PEF.

A further limitation is that the trial was registered retrospectively on ClinicalTrials.gov (NCT03732222) after participant recruitment had already begun in November 2018. Although prospective registration was not yet standard practice nor strictly mandatory for non-pharmacological physiotherapy research in Spain at that time, we acknowledge that retrospective registration deviates from current recommendations of the International Committee of Medical Journal Editors (ICMJE) and may increase the risk of selective reporting. All ethical approvals (protocol code CEI13/01) and written informed consents were obtained prospectively and prior to any participant enrolment, in accordance with the Declaration of Helsinki. The study was registered later to enhance transparency and align with evolving international standards of research integrity.

## 5. Conclusions

In this preliminary randomised sham-controlled trial conducted in healthy young adults, the high-velocity, low-amplitude C3–C4 rotational thrust technique, applied either alone or combined with the diaphragmatic stretch technique, produced statistically significant acute improvements in PEF after a single session. No significant changes were observed in FVC or FEV1. These findings demonstrate that the techniques can acutely influence a dynamic respiratory parameter in asymptomatic individuals without adverse effects on static lung volumes.

The timing of the measurements (immediately and 5 min after the intervention) was intentionally short because the primary aim of this preliminary study was to evaluate the acute physiological effects and safety of the techniques in healthy individuals, rather than their sustained clinical impact. Although our intention was to know the effects (beneficial or detrimental) of using these two techniques together after a single application and in the short term, it is possible that long-term analysis, and/or with more applications, would yield different results. Some studies with these characteristics show that techniques that do not produce positive results in the short term may yield positive results in the mid and long term. Future research with repeated sessions and longer follow-up periods is needed to determine the duration and clinical relevance of the observed effects.

## Figures and Tables

**Table 1:** Population characteristics and baseline spirometry data.

	Total	GC	G1	*p*	G2	*p*	G3	*p*
Age	22.5 ± 5.7	22.1 ± 5.5	23 ± 7.2	0.765	22.5 ± 5.4	0.690	22.6 ± 4.9	0.256
BMI	24.3 ± 13.9	22.7 ± 2.5	23.6 ± 3.5	0.433	28.0 ± 27.0	0.236	23.1 ± 4.2	0.992
Thorax (cm)	91.2 ± 9.5	23.6 ± 3.2	92.5 ± 8.0	0.224	92.2 ± 8.0	0.352	91.0 ± 9.1	0.560
Sex								
Men	62 (40.8)	12 (31.6)	16 (42.1)	0.342	18 (47.4)	0.159	16 (42.1)	0.342
Women	90 (59.2)	26 (68.4)	22 (57.9)		20 (52.6)		22 (57.9)	
Frequency of physical activity								
0 h	39 (25.7)	12 (31.6)	8 (21.1)	0.163	10 (26.3)	0.269	9 (23.7)	0.598
1–5 h	73 (48)	20 (52.6)	17 (44.7)		16 (42.1)		20 (52.6)	
>5 h	40 (26.3)	6 (15.8)	13 (34.2)		12 (31.6)		9 (23.7)	
Intensity of physical activity								
None	39 (25.7)	12 (31.6)	10 (26.3)	0.525	9 (23.7)	0.598	8 (21.1)	0.581
Light-moderate	81 (53.3)	20 (52.6)	18 (47.4)		20 (52.6)		23 (60.5)	
Intense	32 (21.1)	6 (15.8)	10 (26.3)		9 (23.7)		7 (18.4)	
FVC (L)	4.0 ± 0.9	3.8 ± 0.9	4.1 ± 0.8	0.220	4.1 ± 0.8	0.156	3.9 ± 0.9	0.483
FEV1 (L)	2.9 ± 0.9	2.8 ± 0.9	2.9 ± 1.0	0.526	3.0 ± 1.0	0.314	2.8 ± 0.9	0.917
PEF (L/min)	3.7 ± 2.1	3.6 ± 2.2	4.0 ± 2.3	0.374	3.9 ± 2.1	0.278	3.3 ± 1.8	0.967

Quantitative variables shown as mean ± standard deviation. Qualitative variables shown as number (%). The U-Mann–Whitney test was used to compare quantitative variables and the Chi2 test was used to compare qualitative variables. Calculated p vs. control group. CG, control group; G1, stretching group; G2, rotation group; G3, stretching + rotation group; BMI, body mass index.

**Table 2:** Statistical analysis of spirometry values.

		Pre	95% CI	Post	95% CI	*p*	5 Min Post	95% CI	*p*	Friedman *p*
FVC	GC	3.8 ± 0.9	[3.5, 4.1]	3.8 ± 0.9	[3.5, 4.1]	0.166	3.8 ± 0.9	[3.5, 4.1]	0.229	0.815
	G1	4.1 ± 0.8	[3.8, 4.3]	4.1 ± 0.9	[3.8, 4.4]	0.542	4.0 ± 0.9	[3.7, 4.4]	0.470	0.412
	G2	4.1 ± 0.8	[3.8, 4.4]	4.1 ± 0.8	[3.9, 4.3]	0.147	4.1 ± 0.8	[3.8, 4.3]	0.676	0.763
	G3	3.9 ± 0.9	[3.6, 4.2]	4.0 ± 0.9	[3.6, 4.3]	0.664	3.9 ± 0.9	[3.6, 4.3]	0.640	0.825
FEV1	GC	2.8 ± 0.9	[2.5, 3.1]	2.7 ± 1.0	[2.4, 3.1]	0.120	2.7 ± 1.0	[2.4, 3.0]	0.107	0.126
	G1	2.9 ± 1.0	[2.6, 3.3]	2.9 ± 1.0	[2.6, 3.2]	0.777	3.0 ± 0.9	[2.7, 3.3]	0.383	0.444
	G2	3.0 ± 1.0	[2.7, 3.5]	3.0 ± 1.0	[2.7, 3.7]	0.706	3.0 ± 1.0	[2.7, 3.4]	0.739	0.603
	G3	2.8 ± 0.9	[2.5, 3.1]	2.8 ± 1.0	[2.5, 3.1]	0.712	3.0 ± 1.0	[2.5, 3.2]	0.805	0.811
PEF	GC	3.6 ± 2.2	[2.9, 4.3]	3.6 ± 2.1	[2.9, 4.3]	0.643	3.7 ± 2.1	[3.0, 4.4]	0.470	0.822
	G1	4.0 ± 2.3	[3.2, 4.7]	4.1 ± 2.3	[3.3, 4.8]	0.550	4.4 ± 2.3	[3.6, 5.2]	0.133	0.533
	G2	3.9 ± 2.1	[3.2, 4.6]	4.2 ± 2.3	[3.4, 4.9]	0.130	4.3 ± 2.4	[3.5, 5.1]	**0.037**	0.097
	G3	3.3 ± 1.8	[2.8, 3.9]	3.8 ± 2.1	[3.1, 4.5]	**0.026**	3.9 ± 2.1	[3.2, 4.6]	**0.004**	**0.041**

Data shown as mean ± standard deviation. Calculated intragroup *p*-value vs. pre-intervention outcome by Wilcoxon test. Friedman’s test was used to analyse intragroup changes between the three measurement times. CG, control group; G1, stretching group; G2, rotation group; G3, stretching + rotation group. CI = confidence interval. Bold values indicate statistically significant differences (*p* < 0.05).

**Table 3:** Statistical analysis of spirometry values by gender (intra-group analysis).

			Pre	95% CI	Post	95% CI	*p*	5 Min Post	95% CI	*p*	Friedman *p*
Men	FVC	GC	4.7 ± 0.8	[4.3, 5.2]	4.7 ± 0.7	[4.3, 5.1]	0.689	4.7 ± 0.7	[4.2, 5.1]	0.272	0.978
		G1	4.8 ± 0.5	[4.5, 5.1]	4.9 ± 0.5	[4.6, 5.1]	0.05	4.8 ± 0.5	[4.6, 5.1]	0.351	0.087
		G2	4.7 ± 0.7	[4.4, 5.1]	4.7 ± 0.7	[4.4, 5.0]	0.485	4.7 ± 0.6	[4.4, 5.0]	0.556	0.944
		G3	4.7 ± 0.8	[4.3, 5.1]	4.7 ± 0.7	[4.3, 5.1]	0.495	4.7 ± 0.7	[4.3, 5.0]	0.955	0.420
	FEV1	GC	3.6 ± 0.8	[3.1, 4.1]	3.6 ± 0.7	[3.1, 4.0]	0.388	3.5 ± 0.9	[3.0, 4.1]	0.556	0.717
		G1	3.7 ± 0.6	[3.4, 4.0]	3.6 ± 0.7	[3.2, 4.0]	0.816	3.7 ± 0.6	[3.3, 4.0]	0.897	0.444
		G2	3.6 ± 0.9	[3.2, 4.1]	3.5 ± 1.0	[3.0, 4.1]	0.248	3.6 ± 0.9	[3.0, 4.0]	0.695	0.678
		G3	3.5 ± 0.8	[3.1, 4.0]	3.5 ± 1.0	[3.0, 4.0]	0.570	3.4 ± 0.9	[3.0, 3.9]	0.393	0.551
	PEF	GC	4.8 ± 2.5	[3.3, 6.4]	4.8 ± 2.3	[3.3, 6.3]	0.594	4.8 ± 2.5	[3.3, 6.4]	0.929	0.739
		G1	5.4 ± 2.2	[4.2, 6.6]	5.5 ± 2.5	[4.1, 6.8]	0.865	5.6 ± 2.5	[4.2, 6.9]	0.660	0.502
		G2	5.1 ± 2.3	[3.9, 6.2]	5.2 ± 2.6	[3.9, 6.6]	0.687	5.4 ± 2.7	[4.1, 6.7]	0.408	0.459
		G3	4.5 ± 1.9	[3.5, 5.4]	5.0 ± 2.3	[3.7, 6.2]	0.301	5.0 ± 2.2	[3.9, 6.2]	0.05	0.739
Women	FVC	GC	3.4 ± 0.6	[3.2, 3.7]	3.4 ± 0.6	[3.2, 3.6]	0.125	3.4 ± 0.6	[3.1, 3.6]	0.171	0.114
		G1	3.5 ± 0.5	[3.3, 3.8]	3.5 ± 0.6	[3.2, 3.7]	0.537	3.4 ± 0.7	[3.1, 3.8]	0.121	0.566
		G2	3.5 ± 0.4	[3.4, 3.7]	3.5 ± 0.5	[3.3, 3.7]	0.164	3.5 ± 0.4	[3.3, 3.7]	0.758	0.404
		G3	3.4 ± 0.6	[3.2, 3.7]	3.4 ± 0.6	[3.1, 3.7]	0.837	3.4 ± 0.8	[3.2, 3.8]	0.516	0.572
	FEV1	GC	2.4 ± 0.8	[2.1, 2.7]	2.3 ± 0.9	[2.0, 2.7]	0.150	2.3 ± 0.9	[2.0, 2.7]	0.186	0.096
		G1	2.4 ± 0.8	[2.0, 2.7]	2.4 ± 0.7	[2.1, 3.7]	0.833	2.5 ± 0.8	[2.2, 2.9]	0.239	0.800
		G2	2.5 ± 0.7	[2.2, 2.8]	2.5 ± 0.8	[2.2, 2.9]	0.35	2.6 ± 0.8	[2.2, 2.9]	0.296	0.204
		G3	2.3 ± 0.6	[2.0, 2.6]	2.4 ± 0.7	[2.1, 2.7]	0.322	2.4 ± 0.8	[2.1, 2.8]	0.236	0.377
	PEF	GC	3.0 ± 1.9	[2.2, 3.8]	3.0 ± 1.8	[2.3, 3.7]	0.737	3.2 ± 1.8	[2.5, 3.9]	0.467	0.914
		G1	2.9 ± 1.7	[2.2, 3.7]	3.1 ± 1.6	[2.4, 3.8]	0.588	3.4 ± 1.8	[2.6, 4.3]	0.106	0.475
		G2	2.8 ± 1.1	[2.3, 3.3]	3.2 ± 1.3	[2.6, 3.8]	**0.018**	3.4 ± 1.8	[2.6, 4.2]	**0.025**	0.092
		G3	2.5 ± 1.2	[2.0, 3.1]	3.0 ± 1.6	[2.3, 3.7]	**0.012**	3.1 ± 1.7	[2.4, 3.9]	**0.038**	**0.041**

Data shown as mean ± standard deviation. Calculated intragroup *p*-value vs. pre-intervention outcome by Wilcoxon test. Friedman’s test was used to analyse intragroup changes between the three measurement times. CG, control group; G1, stretching group; G2, rotation group; G3, stretching + rotation group. CI = confidence interval. Bold values indicate statistically significant differences (*p* < 0.05).

## Data Availability

In order to enhance transparency and ensure compliance with best scientific practices, we would like to clarify that the dataset supporting the findings of this study is publicly available in Zenodo. The dataset can be accessed without restrictions via the following DOI: https://doi.org/10.5281/zenodo.20747586. All data are fully anonymized and comply with ethical standards.

## Abbreviations

The following abbreviations are used in this manuscript:

FVC	Forced vital capacity
FEV1	Forced expiratory volume in 1 s
PEF	Peak expiratory flow
COPD	Chronic Obstructive Pulmonary Disease
PR	Rehabilitation protocol
OMT	Osteopathic manual treatment
BMI	Body Mass Index
